# Supplementary material for: The impact of the COVID-19 pandemic on children with medical complexity
Source: BMC Pediatr. 2022 Aug 23;22:496. doi: 10.1186/s12887-022-03549-y (PMC9398046; doi:10.1186/s12887-022-03549-y)
Supplement: Supplementary file 3 — Additional file 3. [file 12887_2022_3549_MOESM3_ESM.docx]

| A Consensus-Based Checklist for Reporting of Survey Studies (CROSS) (Sharma et al; 2021) | Completed  (Y: yes; N: no; n/a: not applicable |
| --- | --- |
| 1. Title and abstract |  |
| 1. State the word “survey” along with a commonly used term in title or abstract to introduce the study’s design 2. Provide an informative summary in the abstract, covering background, objectives, methods, findings/results, interpretation/discussion, and conclusions. | Yes  Yes |
| 1. Introduction and background |  |
| 1. Provide a background about the rationale of study, what has been previously done, and why this survey is needed. | Yes |
| 1. Purpose and aim |  |
| 1. Identify specific purposes, aims, goals, or objectives of the study. | Yes |
| 1. Methods and study design |  |
| 1. Specify the study design in the “Methods” section with a commonly used term (e.g., cross-sectional or longitudinal). | n/a (involved all paediatricians enrolled in CPSP at single time) |
| 1. Data collection methods |  |
| 1. Describe the questionnaire (e.g., number of sections, number of questions, number and names of instruments used). 2. Describe all questionnaire instruments that were used in the survey to measure particular concepts. Report target population, reported validity and reliability information, scoring/classification procedure, and reference links (if any). 3. Provide information on pretesting of the questionnaire, if performed. Report the method of pretesting, number of times questionnaire was pre-tested, number and demographics of participants used for pretesting, and the level of similarity of demographics between pre-testing participants and sample population. 4. Questionnaire, if possible, should be fully provided (in the article, or as appendices or as an online supplement). | Yes  n/a  No (Reviewed internally by steering committee and by CPSP)  Yes |
| 1. Sample characteristics |  |
| 1. Describe the study population (i.e., background, locations, eligibility criteria for participant inclusion in survey, exclusion criteria). 2. Describe the sampling techniques used (e.g., single stage or multistage sampling, simple random sampling, stratified sampling, cluster sampling, convenience sampling). Specify the locations of sample participants whenever clustered sampling was applied 3. Provide information on sample size, along with details of sample size calculation. 4. Describe how representative the sample is of the study population (or target population if possible), particularly for population-based surveys. | Yes  Yes  n/a  Yes |
| 1. Survey administration |  |
| 1. Provide information on modes of questionnaire administration. 2. Provide information of survey’s timeframe, such as periods of recruitment, exposure, and follow-up days. 3. Provide information on the entry process:–>For non-web-based surveys, provide approaches to minimize human error in data entry.–>For web-based surveys, provide approaches to prevent “multiple participation” of participants. | Yes  Yes  Yes (each participant receives a unique and coded link preventing them from completing the survey twice. Once they have completed the survey, the link they received becomes inactive) |
| 1. Study preparation |  |
| 1. Describe any preparation process before conducting the survey (e.g., interviewers’ training process, advertising the survey). | Yes (conducted using CPSP infrastructure) |
| 1. Ethical considerations |  |
| 1. Provide information on ethical approval for the survey if obtained, including informed consent, institutional review board [IRB]approval, Helsinki declaration, and good clinical practice [GCP]declaration (as appropriate). 2. Provide information about survey anonymity and confidentiality and describe what mechanisms were used to protect unauthorized access. | Yes (was reviewed by CPSP board)  Yes |
| 1. Statistical analysis |  |
| 1. Describe statistical methods and analytical approach. Report the statistical software that was used for data analysis. 2. Report any modification of variables used in the analysis, along with reference (if available). 3. Report details about how missing data was handled. Include rate of missing items, missing data mechanism (i.e., missing completely at random [MCAR], missing at random [MAR], or missing not at random [MNAR]), and methods used to deal with missing data (e.g., multiple imputation). 4. State how non-response error was addressed. 5. For longitudinal surveys, state how loss to follow-up was addressed. 6. Indicate whether any methods such as weighting of items or propensity scores have been used to adjust for non-representativeness of the sample. 7. Describe any sensitivity analysis conducted. | Yes  n/a  Yes  Yes  n/a  No  No |
| 1. Respondent characteristics |  |
| 1. Report numbers of individuals at each stage of the study. Consider using a flow diagram, if possible. 2. Provide reasons for non-participation at each stage, if possible. 3. Report response rate, present the definition of response rate or the formula used to calculate response rate. 4. Provide information to define how unique visitors are determined. Report number of unique visitors | n/a  n/a  Yes  n/a |
| 1. Descriptive results |  |
| 1. Provide characteristics of study participants, as well as information on potential confounders and assessed outcomes | Yes |
| 1. Main findings |  |
| 1. Give unadjusted estimates and, if applicable, confounder-adjusted estimates along with 95% confidence intervals and p-values. 2. For multivariable analysis, provide information on the model building process, model fit statistics, and model assumptions (as appropriate). 3. Provide details about any sensitivity analysis performed. If there are considerable amount of missing data, report sensitivity analyses comparing the results of complete cases with tha tof the imputed dataset (if possible). | n/a  n/a  n/a |
| 1. Discussion / Limitations |  |
| 1. Discuss the limitations of the study, considering sources of potential biases and imprecisions, such as non-representativeness of sample, study design, important uncontrolled confounders | Yes |
| 1. Interpretations |  |
| 1. Give a cautious overall interpretation of results, based on potential biases and imprecisions and suggest areas for future research. | Yes |
| 1. Generalizability |  |
| 1. Discuss the external validity of the results. | Yes |
| 1. Role of the funding sources |  |
| 1. State whether any funding organization has had any roles in the survey’s design, implementation, and analysis. | Yes |
| 1. Conflict of interest |  |
| 1. Declare any potential conflict of interest. | Yes |
| 1. Acknowledgements |  |
| 1. Provide names of organizations/persons that are acknowledged along with their contribution to the research. | Yes |
